# The Relation Between Health and Earnings in Self-Employment

**DOI:** 10.3389/fpsyg.2020.00801

**Published:** 2020-05-26

**Authors:** Jolanda Hessels, Cornelius A. Rietveld, Peter van der Zwan

**Affiliations:** ^1^Department of Applied Economics, Erasmus School of Economics, Erasmus University Rotterdam, Rotterdam, Netherlands; ^2^Erasmus University Rotterdam Institute for Behavior and Biology, Erasmus University Rotterdam, Rotterdam, Netherlands; ^3^Department of Business Studies, Leiden Law School, Leiden University, Leiden, Netherlands

**Keywords:** earnings, health, HILDA data, human capital, self-employment

## Abstract

Multiple studies have shown that, on average, the self-employed are healthier than wage workers. The link between the health of self-employed individuals and their financial performance in terms of earnings is, however, less understood. Based on human capital theory, we expect a positive link between health and earnings among the self-employed. For two reasons we expect the relationship between health and earnings to be stronger for the self-employed than for wage workers. First, the self-employed can more easily adapt their production activities such that they yield the highest returns to their human capital, including their health. Second, in the short term, the earnings of the self-employed are more dependent on the ability to work than the wages of wage workers. Our empirical analysis draws on data from the Household, Income and Labor Dynamics in Australia (HILDA) survey, a longitudinal dataset (2001–2017). Our outcome variable is an individual’s total income derived from wage work and/or running a business. Health is measured using multi-item constructs for *General health*, *Physical health*, and *Mental health* from the Short Form Health Survey (SF-36). We distinguish between wage workers and self-employed individuals with and without employees. Fixed-effects regressions reveal a significant positive relationship between health and earnings in self-employment as well as in wage work. As expected, this relationship is significantly stronger in self-employment than in wage work (for *General health* and *Physical health*, but not for *Mental health*). The latter result holds particularly for self-employment without employees. We provide evidence that the higher returns can be partly explained by the fact that the earnings in self-employment are more dependent on the ability to work (as proxied by the number of working hours) than earnings in wage work. We also find a negative relationship between health and job termination. Again, this relationship is stronger for the self-employed (without employees) than for wage workers (for *General health* and *Mental health*, but not for *Physical health*).

## Introduction

The self-employed represent a considerable portion of the labor force in developed countries. By setting up and running businesses, the self-employed contribute to the creation of employment for their own and for others (Van Praag and Versloot, 2007; De Wit and De Kok, 2014). Hence, governments recognize self-employment as pivotal for achieving growth (Audretsch and Keilbach, 2004; Carree and Thurik, 2010; Koellinger and Thurik, 2012), and therefore they actively support self-employment (European Commission, 2020). The occupational notion of self-employment stresses that self-employed individuals own and manage their business for their own account and risk (Wennekers and Thurik, 1999). In doing so, they can set up their production activities such that they yield the highest returns to their assets, including their human capital (Van Praag et al., 2013; Hessels et al., 2020). Indeed, there have been many studies investigating the returns to self-employment in terms of earnings (Hamilton, 2000; Sorgner et al., 2017). The present study analyses the relationship between a specific dimension of human capital – an individual’s health status – and earnings.

An increasing number of studies stresses the importance of health for self-employment. This is because the self-employed work in a complex and uncertain environment, work long hours, and have to perform a wide range of tasks (Hessels et al., 2018). Good health is important to deal with the challenges and difficulties that come with running a business (Gielnik et al., 2012; Hessels et al., 2018). Early studies found ambiguous associations between health and self-employment (Quinn, 1980; Fredland and Little, 1981; Curran and Burrows, 1989; Parker, 2004), but more recent studies generally report a positive association between self-employment (versus wage work) and health (Tetrick et al., 2000; Bradley and Roberts, 2004; Stephan and Roesler, 2010; Castellano and Punzo, 2013; Castellano et al., 2016; Toivanen et al., 2019). Although switching to self-employment out of unemployment and wage work may have a short-term positive effect on health (Nikolova, 2019), the selection of healthy individuals into self-employment seems to prevail such contextual effects in explaining the positive relation between self-employment and health (Yoon and Bernell, 2013; Rietveld et al., 2015). The reason is that in self-employment, there are not only factors that contribute positively to health but also factors that may affect health adversely (Torrès, 2012; Torrès and Thurik, 2019), such as the inherent risky nature of self-employment and its associated uncertainties (Buttner, 1992; Dahl, 2011).

In addition to research on possible health differences between the self-employed and wage workers, recent studies have started to focus on the relationship between health and financial performance in self-employment (Hatak and Zhou, 2019), usually measured in terms of earnings (Parker, 2018). However, the exact relationship between health and performance in self-employment has remained largely unidentified. Specifically, it is not clear from previous research whether and why the health–earnings relationship is different for individuals in self-employment and individuals in wage work. In the present study, we provide a theoretical explanation for why we can expect a stronger relationship between health and earnings in self-employment as compared to wage work and we perform a direct empirical test of this prediction.

The theoretical explanation we put forward in this study originates from human capital theory. Human capital is the set of skills, knowledge, and social and personality attributes that constitute the ability to perform labor and to produce economic value. Prior self-employment studies suggest that human capital in terms of cognitive ability (Hartog et al., 2010) and formal education (Robinson and Sexton, 1994; Van Praag et al., 2013; Hessels et al., 2020) results in comparatively high returns in terms of earnings in self-employment relative to wage work. The relation between earnings and health, as another important element of human capital (Becker, 1962; Hatak and Zhou, 2019), has so far received little attention in the self-employment literature (Rietveld et al., 2016; Hatak and Zhou, 2019). In line with human capital theory, we expect a positive relationship between health and earnings, not only for wage workers (Pelkowski and Berger, 2004), but also for the self-employed. Moreover, we expect that the positive relationship between health and earnings is stronger for the self-employed than for wage workers. First of all, earlier studies stress that the self-employed can adapt their production activities more easily than wage workers such that they yield the highest returns to their assets (Van Praag et al., 2013; Hatak and Zhou, 2019). Second, the earnings in self-employment, at least in the short run, are more dependent on the ability to work than the wages of wage workers (Rietveld et al., 2015). We therefore expect that good health boosts earnings in self-employment more than earnings in wage work, but that ill health reduces earnings more drastically in self-employment than in wage work.

We investigate the empirical validity of our expectations by analyzing data from the Household, Income and Labor Dynamics in Australia (HILDA) survey. HILDA is a household-based longitudinal dataset that has been in existence since 2001. We use information for the period 2001–2017, and our analysis sample comprises 111,495 person-year observations (from 17,701 distinct individuals). Fixed-effects regressions are performed to take account of the longitudinal structure of our dataset, and they reveal a significant positive relationship between health and earnings for both wage workers and self-employed individuals. Moreover, moderation analyses show that the relation between health and earnings is indeed stronger in self-employment than in wage work. We also find a more negative relationship between health and job termination for the self-employed than for wage workers. In our analyses, we also distinguish between two types of self-employment, i.e., self-employment without employees and with employees. This distinction is important to make, because the two groups of self-employed workers have been shown to differ in (some dimensions of) health (Beutell et al., 2014). For example, the distinction between the two types of self-employment is relevant in explaining differences in perceived stress (Hessels et al., 2017), life satisfaction (Johansson Sevä et al., 2016), and work pressure (Blanchflower, 2004). Our analyses show that the main results hold in particular for the self-employed without employees. For them, ill health is particularly harmful as there are no other people who can take over tasks in case of reduced ability to work.

The present study contributes to the growing stream of research on the relation between self-employment and health, and makes three specific contributions to this literature. First, based on the theoretical premise of the human capital literature, we show that the relationship between health and financial performance holds both for wage workers and the self-employed. Second, although many individuals are attracted to self-employment by features such as the relatively high level of decision of authority (Benz and Frey, 2008) and the possibility of high earnings (Taylor, 2004), our findings imply that the earnings of the self-employed are particularly sensitive to health deteriorations. As such, our study contributes to a more nuanced picture of the outcomes of a career in self-employment. Third, following a recent stream of studies in the self-employment literature, we distinguish between self-employed individuals with and without employees. Our results show that the strength of the relation between health and earnings is different for these two occupational groups. The heterogeneity between the two groups is important from a policy perspective, given the steady increase in the number of self-employed individuals without employees in most developed countries (Van Stel and van der Zwan, 2019).

## Materials and Methods

### Sample

The Household, Income and Labor Dynamics in Australia (HILDA) survey is a household-based longitudinal dataset that exists since 2001. We use the HILDA survey in this study because it contains detailed longitudinal information about health, occupational status, and earnings. We use data covering the period 2001–2017. We refer to Summerfield et al. (2019) for more detailed information about the survey.

### Variables

#### Dependent Variable

Our dependent variable *Earnings* reflects the sum of an individual’s gross wage/salary income and his/her business income per year. Negative and zero values are not considered. The variable is logarithmically transformed because of its skewness.

#### Independent Variables

Our variables capturing health are constructed using items from the Short Form Health Survey (SF-36) questionnaire (Ware and Sherbourne, 1992). The SF-36 questionnaire distinguishes between eight scales in total, which are averages of separate items in the questionnaire (Ware and Sherbourne, 1992). To provide a comprehensive analysis of the relationship between health and earnings, we use the scales for general health, bodily pain, and mental health in our study. The *General health* variable is constructed using five items: (i) In general, would you say your health is … Excellent; Very good; Good; Fair; or Poor, (ii) I seem to get sick a little easier than other people (True; Not true), (iii) I am as healthy as anybody I know (True; Not true), (iv) I expect my health to get worse (True; Not true), and (v) My health is excellent (True; Not true). Higher values reflect better general health (Ware et al., 2000); Cronbach alpha equals 0.80. *Physical health* was measured with the following two items: (i) How much bodily pain have you had during the past 4 weeks? (No bodily pain; Very mild; Mild; Moderate; Severe; Very severe), and (ii) During the past 4 weeks, how much did pain interfere with your normal work (including both work outside the home and housework)? (Not at all; Slightly; Moderately; Quite a bit; Extremely). Again, the values are transformed in such a way that higher values reflect better physical health (Ware et al., 2000); Cronbach alpha equals 0.63. *Mental health* was measured with the following four items: How much of the time during the past 4 weeks … (i) … have you been a very nervous person? (ii) … have you felt so down in the dumps that nothing could cheer you up? (iii) … have you felt calm and peaceful? (iv) … have you felt downhearted and blue? and (v) … have you been a happy person? Higher values reflect better mental health (Ware et al., 2000); Cronbach alpha equals 0.83. These three variables capturing health have been standardized to have mean zero and standard deviation one in the analysis sample.

#### Moderator Variables

The binary variable *Self-employment* distinguishes individuals in self-employment (1) from individuals in wage work (0). In further analyses, we use the two binary variables *Self-employment with employees* and *Self-employment without employees* (for both variables the reference category comprises wage workers) to distinguish self-employed individuals with and without employees. The variables are derived from a question asking individuals whether, at any time at all during the last 7 days, they did any work in a job or a business. In follow-up questions individuals reveal whether they worked for an employer for wages or salary, or whether they worked in their own business, without or with employees. We focus on an individual’s main job. That is, if a respondent says (s)he works in more than one job, the job is selected where (s)he gets the most pay from.

#### Control Variables

In our regressions, we control for the demographic variables *Age* (in years; only individuals between 18 and 64 years are included in our analysis), *Age squared* and *Education* (total years of completed schooling)^1^. Age and age squared have been included in numerous earlier studies on entrepreneurial earnings (e.g., Taylor, 2001); the same holds for educational attainment (Hamilton, 2000; Van Praag et al., 2013). *Marital status* (dummy variables for registered marriages and “separated/divorced/widowed”; “not married” is the reference category; see Wong, 1986; Hamilton, 2000) and *Children* (the number of own resident children) have also been included as control variables (Sorgner et al., 2017). Furthermore, we control for the work-related characteristics *Tenure in current business/job* (the total number of years worked in the current business for the self-employed or in the current job for the wage workers, logarithmically transformed) and *Tenure occupation* (the total number of years worked in the same current occupation – wage work or self-employment – logarithmically transformed). Tenure is commonly included in earnings regressions (Hamilton, 2000). We also control for living area (Burke et al., 2000)^2^, and we include year and industry dummies (one-digit industry classification^3^; 19 industries are distinguished), see also Hvide (2009).

### Empirical Strategy

We perform linear fixed-effects regressions with *Earnings* (in logarithms) as the dependent variable. Time-invariant factors are controlled for in fixed-effects regressions, and, hence, our regressions exploit the within-person variation over time (Hajek and König, 2016)^4^. The estimated coefficients inform us about the percentual change in the dependent variable as the result of a one-unit change (=1 standard deviation change because the independent variables have been standardized) in the independent variable. To further deal with the possibility of reverse causality bias, we use earnings in the subsequent period (one year ahead) as our dependent variable, whereas all independent and control variables are from the current period. In doing so, we follow other studies in this area (e.g., Hatak and Zhou, 2019). To allow for a different relationship between health and earnings for different type of workers we include interaction terms between our health measures and (1) our binary variable *Self-employment*; and (2) our binary variables *Self-employment with employees* and *Self-employment without employees*. Cluster-robust standard errors are used in all our regressions.

## Results

### Main Results

Table 1 provides an overview of all variables included in the analysis, together with some descriptive statistics. Importantly, from the 111,495 individual-year observations in our analysis sample, 15,773 come from individuals in self-employment (14.1%) and 95,722 from wage workers (85.9%).

**TABLE 1:** Descriptive statistics of the analysis sample.

Variable	Min.	Max.	Wage work		Self-employment	
			Mean	SD	Mean	SD
Earnings (logarithm)	0	14.34	10.67	0.84	10.47	1.17
General health	−3.92	1.51	−0.01	1.00	0.03	1.00
Physical health	−3.75	1.07	0.01	0.99	−0.09	1.03
Mental health	−4.83	1.57	−0.01	1.01	0.09	0.96
Self-employment without employees	0	1			0.59	0.49
Self-employment with employees	0	1			0.41	0.49
Working hours (weekly; logarithm)	−4.61	5.01	3.51	0.53	3.55	0.72
Age	18	64	38.80	12.39	45.31	10.60
Education	8	17	12.73	2.11	12.51	2.11
Not married	0	1	0.41	0.49	0.23	0.42
Married	0	1	0.50	0.50	0.69	0.46
Separated/divorced/widowed	0	1	0.09	0.29	0.08	0.27
Children	0	5	0.84	1.09	1.12	1.23
Tenure business/job (logarithm)	−3.95	3.95	1.07	1.51	1.72	1.35
Tenure SE/wage work (logarithm)	−3.95	3.95	1.40	1.52	2.17	1.28

*SD, standard deviation. Table is based on 111,495 observations (17,701 distinct individuals), of which 15,773 refer to self-employment (3,853 distinct self-employed individuals) and 95,722 to wage work (16,242 distinct wage workers). General health, Physical health, and Mental health have been standardized (mean 0, standard deviation 1 in our analysis sample). “Not married” serves as a reference category in our regressions. Values larger than 5 for Children have been recoded to 5. Descriptive statistics for the living areas, industries, and wave dummies are available upon request from the authors.*

Table 2 shows the results of three fixed-effects regressions. The first column focuses on the relationship between *General health* and *Earnings* for the self-employed and wage workers. The second column zooms in on *Physical health*; the third column includes our *Mental health* measure. The results in the first column of Table 2 reveal that the positive relationship between *General health* and *Earnings* is significantly stronger in self-employment than in wage work. While a one-standard deviation increase in *General health* (which equals an increase of 18 points on the original scale ranging from 0 to 100) is associated with a 1.1%-increase in earnings in wage work, this increase amounts to 3.9% in self-employment. For *Physical health* (the second column) we retrieve similar results. That is, a one-standard deviation increase (21 points on the original scale) in *Physical health* is associated with a 1.5%-increase in earnings in wage work, and a 4.2%-increase in self-employment. For *Mental health* we do not find a significantly stronger relationship between health and earnings in self-employment. However, we do find that a one-standard deviation increase (16 points on the original scale) in the *Mental health* measure is associated with a 1.3%-increase in earnings in wage work, and a 1.0%-increase in self-employment.

**TABLE 2:** Fixed-effects regressions with *Earnings* (in logarithms) in the subsequent period as the dependent variable.

	General health (1)		Physical health (2)		Mental health (3)	
	Coefficients	SE	Coefficients	SE	Coefficients	SE
Health	0.011^∗∗^	0.004	0.015^∗∗∗^	0.003	0.013^∗∗∗^	0.003
Self-employment	–0.319^∗∗∗^	0.019	–0.317^∗∗∗^	0.019	–0.318^∗∗∗^	0.019
Health × Self-employment	0.028^∗^	0.013	0.027^∗∗^	0.010	–0.003	0.011
Age	0.163^∗∗∗^	0.003	0.163^∗∗∗^	0.003	0.164^∗∗∗^	0.003
Age squared	–0.001^∗∗∗^	0.000	–0.001^∗∗∗^	0.000	–0.001^∗∗∗^	0.000
Education	0.129^∗∗∗^	0.006	0.129^∗∗∗^	0.006	0.129^∗∗∗^	0.006
Married	–0.089^∗∗∗^	0.011	–0.089^∗∗∗^	0.011	–0.089^∗∗∗^	0.011
Separated/divorced/widowed	–0.061^∗∗∗^	0.017	–0.062^∗∗∗^	0.017	–0.059^∗∗∗^	0.017
Children	–0.075^∗∗∗^	0.005	–0.076^∗∗∗^	0.005	–0.075^∗∗∗^	0.005
Tenure business/job	0.032^∗∗∗^	0.002	0.033^∗∗∗^	0.002	0.033^∗∗∗^	0.002
Tenure SE/wage work	0.013^∗∗∗^	0.002	0.013^∗∗∗^	0.002	0.013^∗∗∗^	0.002
Observations	111,495		111,495		111,495	
Individuals	17,701		17,701		17,701	
*R*^2^ (within)	0.21		0.21		0.21	

*^∗^p < 0.05; ^∗∗^p < 0.01; and ^∗∗∗^p < 0.001. SE, cluster-robust standard error. Sector, wave, and state dummies (and the intercept) are included; the corresponding estimates are available upon request from the authors.*

Table 3 distinguishes between self-employment without and with employees. Our sample contains 2,732 self-employed individuals without employees (8,433 person-year observations) and 1,721 self-employed individuals with employees (5,921 person-year observations). The results in Table 3 show that the main result for *General health* (Table 2) applies to the self-employed without employees only (a Wald χ^2^-test for the equivalence of the coefficients of the two self-employment groups gives *p* = 0.05), while we find a stronger relationship between *Physical health* and *Earnings* for both groups of self-employed workers compared to wage workers. A Wald χ^2^-test (*p* = 0.80) indicates that an increase in the *Physical health* measure is associated with the same change in earnings for each type of self-employment, compared to wage work. For *Mental health*, we do not find a significantly stronger relationship with *Earnings* for either type of self-employment.

**TABLE 3:** Fixed-effects regressions with *Earnings* (in logarithms) in the subsequent period as the dependent variable.

	General health (1)		Physical health (2)		Mental health (3)	
	Coefficients	SE	Coefficients	SE	Coefficients	SE
Health	0.012^∗∗^	0.004	0.015^∗∗∗^	0.003	0.014^∗∗∗^	0.003
Self-employment without employees	–0.407^∗∗∗^	0.022	–0.406^∗∗∗^	0.022	–0.408^∗∗∗^	0.022
Self-employment with employees	–0.171^∗∗∗^	0.023	–0.170^∗∗∗^	0.023	–0.170^∗∗∗^	0.023
Health × Self-employment without employment	0.042^∗^	0.018	0.028^∗^	0.014	0.006	0.015
Health × Self-employment with employment	0.0002	0.017	0.033^∗^	0.014	–0.021	0.015
Age	0.164^∗∗∗^	0.003	0.163^∗∗∗^	0.003	0.164^∗∗∗^	0.003
Age squared	–0.001^∗∗∗^	0.000	–0.001^∗∗∗^	0.000	–0.001^∗∗∗^	0.000
Education	0.128^∗∗∗^	0.006	0.128^∗∗∗^	0.006	0.128^∗∗∗^	0.006
Married	–0.087^∗∗∗^	0.011	–0.087^∗∗∗^	0.011	–0.087^∗∗∗^	0.011
Separated/divorced/widowed	–0.059^∗∗∗^	0.017	–0.059^∗∗∗^	0.017	–0.056^∗∗∗^	0.017
Children	–0.079^∗∗∗^	0.005	–0.080^∗∗∗^	0.005	–0.079^∗∗∗^	0.005
Tenure business/job	0.032^∗∗∗^	0.002	0.032^∗∗∗^	0.002	0.032^∗∗∗^	0.002
Tenure SE/wage work	0.012^∗∗∗^	0.002	0.013^∗∗∗^	0.002	0.012^∗∗∗^	0.002
Observations	110,076		110,076		110,076	
Individuals	17,573		17,573		17,573	
*R*^2^ (within)	0.21		0.21		0.21	

*Distinction between self-employed individuals without and with employees. ^∗^p < 0.05; ^∗∗^p < 0.01; and ^∗∗∗^p < 0.001. SE, cluster-robust standard error. Sector, wave, and state dummies (and the intercept) are included; the corresponding estimates are available upon request from the authors. Information about having employees is unknown for 1,419 observations.*

**FIGURE 1:**
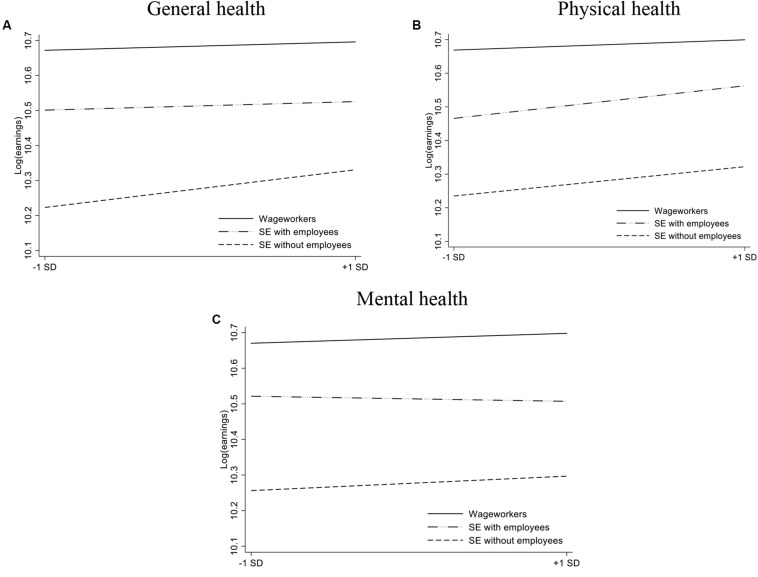
The relationship between our health measures (Panel **A**: General health, Panel **B**: Physical health, Panel **C**: Mental Health) and *Earnings* for wage workers, self-employed individuals without employees, and self-employed individuals with employees (based on the regression results in Table 3).

Interaction plots based on the regressions in Table 3 are displayed in Figure 1. The figures show the predicted values of the dependent variable *Earnings* for the values of the standardized health variables (with −1 and +1 chosen as minimum and maximum values). Figure 1A (*General health*) shows relatively flat lines for wage work and self-employment with employees, and a steeper line for self-employment without employees. Figure 1B (*Physical health*) shows steeper lines for both self-employment groups compared with wage work, and Figure 1C (*Mental health*) displays three lines with a relatively equal slope.

### Additional Results

#### Other Labor Market Outcomes

Two other labor market outcomes frequently analyzed in the health economics literature are the number of working hours and the transition into unemployment (Lenhart, 2019). To complement the main analyses, we focus on the number of working hours per week first. We find that the number of working hours partly explains the higher returns to health in terms of earnings. That is, after adding the number of working hours to the specification in Table 2, we observe smaller interaction coefficients in the regressions for *General health* and *Physical health*. The results are displayed in Table 4 Panel A. Panel B distinguishes between self-employment without and with employees, and we observe reduced coefficients for the interaction term *Health* × *Self-employment without employees*. Thus, in line with our reasoning, the stronger positive relationship between health (generally and physically) and earnings in self-employment compared to wage work is partly explained by the number of working hours, i.e., the ability to work^5^.

**TABLE 4:** Fixed-effects regressions with *Earnings* (in logarithms) in the subsequent period as the dependent variable and *Working hours* as an additional control variable.

Panel A						
	General health (1)		Physical health (2)		Mental health (3)	
	Coefficients	SE	Coefficients	SE	Coefficients	SE
Health	0.009^∗∗^	0.003	0.014^∗∗∗^	0.002	0.012^∗∗∗^	0.003
Self-employment	–0.269^∗∗∗^	0.018	–0.267^∗∗∗^	0.018	–0.268^∗∗∗^	0.018
Health × Self-employment	0.018	0.013	0.021^∗^	0.010	–0.006	0.011
Observations	111,495		111,495		111,495	
Individuals	17,701		17,701		17,701	
*R*^2^ (within)	0.30		0.30		0.30	

**Panel B**						

Health	0.010^∗∗^	0.003	0.015^∗∗∗^	0.002	0.013^∗∗∗^	0.003
Self-employment without employees	–0.327^∗∗∗^	0.020	–0.326^∗∗∗^	0.020	–0.328^∗∗∗^	0.020
Self-employment with employees	–0.171^∗∗∗^	0.023	–0.169^∗∗∗^	0.023	–0.169^∗∗∗^	0.023
Health × Self-employment without employment	0.030	0.017	0.025	0.014	–0.001	0.014
Health × Self-employment with employment	0.001	0.017	0.026	0.015	–0.019	0.016
Observations	110,076		110,076		110,076	
Individuals	17,573		17,573		17,573	
*R*^2^ (within)	0.21		0.21		0.21	

*^∗^p < 0.05; ^∗∗^p < 0.01; and ^∗∗∗^ p < 0.001. SE, cluster-robust standard error. Control variables (including working hours) are included; the corresponding estimates are available upon request from the authors. Panel B distinguishes between the self-employed without and with employees. Information about having employees is unknown for 1,419 observations.*

**FIGURE 2:**
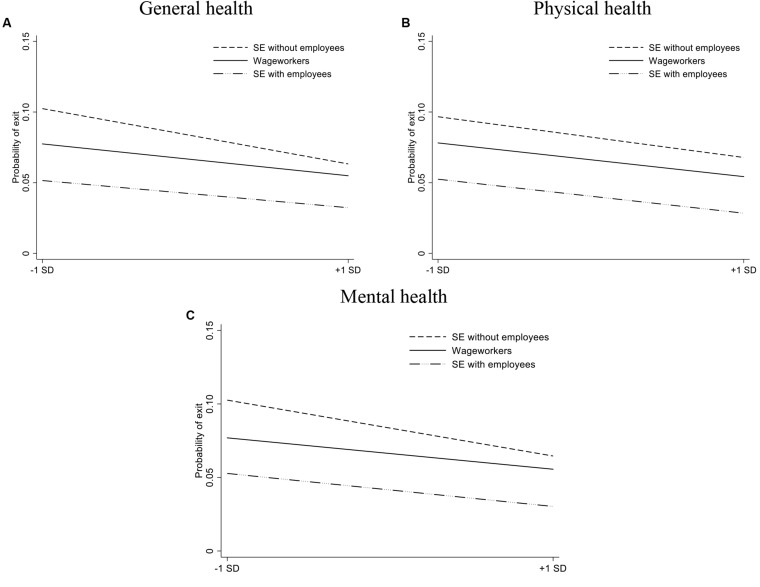
The relationship between our health measures (Panel **A**: General health, Panel **B**: Physical health, Panel **C**: Mental Health) and *Earnings* for wage workers, self-employed individuals without employees, and self-employed individuals with employees (based on the regressions results in Table 5).

Second, we focus on the probability that an individual active in the labor market – in self-employment or in wage work – becomes unemployed or moves out of the labor force in the subsequent time period (exit between *t* and *t* + 1). Binary logistic regressions with *Exit* as dependent variable (Allison, 1982) show that there is a particularly strong negative relationship between health and *Exit* for the self-employed without employees (compared with wage workers) in case of *General health* and *Mental health* (Table 5). Hence, while poorer health is associated with a higher probability of leaving one’s current job, we note that this association is stronger for the self-employed (without employees) compared with wage workers. The interaction plots depicting these relationships are provided in Figure 2.

**TABLE 5:** Binary logistic regressions with *Exit* as the dependent variable.

Panel A						
	General health (1)		Physical health (2)		Mental health (3)	
	Coefficients	SE	Coefficients	SE	Coefficients	SE
Health	–0.189^∗∗∗^	0.012	–0.200^∗∗∗^	0.012	–0.178^∗∗∗^	0.012
Self-employment	0.0002	0.039	–0.010	0.040	–0.001	0.039
Health × Self-employment	–0.092^∗∗^	0.032	–0.034	0.031	–0.103^∗∗^	0.032
Observations	113,096		113,096		113,096	
Individuals	17,749		17,749		17,749	

**Panel B**						

Health	–0.189^∗∗∗^	0.012	–0.201^∗∗∗^	0.012	–0.179^∗∗∗^	0.012
Self-employment without employees	0.237^∗∗∗^	0.045	0.243^∗∗∗^	0.046	0.246^∗∗∗^	0.044
Self-employment with employees	–0.507^∗∗∗^	0.074	–0.563^∗∗∗^	0.079	–0.531^∗∗∗^	0.074
Health × Self-employment without employment	−0.082^∗^	0.036	0.001	0.036	−0.082^∗^	0.037
Health × Self-employment with employment	–0.059	0.067	–0.125	0.066	–0.116	0.066
Observations	111,607		111,607		111,607	
Individuals	17,634		17,634		17,634	

*^∗^p < 0.05; ^∗∗^p < 0.01; and ^∗∗∗^p < 0.001. SE, cluster-robust standard error. Control variables are included; the corresponding estimates are available upon request from the authors. Panel B distinguishes between the self-employed without and with employees. Information about having employees is unknown for 1,489 observations. The analysis sample in slightly larger than the sample used for the regressions reported in Table 2, because some individuals reported employment information without providing information about their earnings.*

#### Health Shocks

As a robustness analysis, we analyze the validity of our results in a set-up in which we “match” individuals who experience a negative health shock with individuals who do not experience a health shock and remain in good health^6^. Hence, apart from this health shock, the two groups are similar in terms of the control variables listed above. By using this type of so-called propensity score matching, we “isolate” the impact of a health shock on *Earnings*. Earlier studies have used similar approaches to infer causal relationships between health and relevant outcomes (García-Gómez, 2011; Lan et al., 2019). In line with the main findings reported in Table 2, we find again that the effect on *Earnings* for those experiencing a (negative) health shock is much stronger in self-employment than in wage work. Again, we find that these results hold for *General Health* and *Physical health* but not for *Mental health*. Estimation results are available upon request from the authors.

## Discussion

Our analyses show that an increase in general health is associated with an increase in earnings in wage work. Notably, the increase in earnings resulting from the same increase in health is more than twice as large in self-employment and thus the difference between the two groups of workers is substantial. We note that this pattern of a stronger relationship between health and earnings is very comparable for general health and our measure of physical health, while the returns to health are similar in self-employment and wage work in case of mental health. Additional analyses show that being healthy generally and mentally is more important for the self-employed (without employees) than for wage workers in terms of remaining in their present job. While earlier research has shown that the presence of depressive symptoms may precede a switch out of self-employment (Hessels et al., 2018), the present analysis stresses that the effect of mental health on job termination is larger in self-employment than in wage work.

Our additional analyses show that these results can be partially explained by the notion that, at least in the short term, earnings in self-employment are more dependent on the ability to work (as proxied by the number of working hours) than earnings in wage work. Besides, decision authority, an essential difference between the occupations of the self-employed and wage workers (Hébert and Link, 1989; Hundley, 2001; Stephan and Roesler, 2010), may partially explain the stronger relationship between health and financial performance for the self-employed compared to wage workers. Decision authority at work makes individuals feel responsible for work outcomes (Hackman and Oldham, 1976) and has been associated with improved work performance (Bond and Bunce, 2001). For the self-employed, it also makes that they can more easily adapt their production activities such that they yield higher returns to their human capital assets (Van Praag et al., 2013).

We note that the empirical results are based on the analysis of an Australian dataset, and this raises the question as to whether the revealed relationships between health and earnings are specific to Australia or applicable to other countries as well. A related study about the relationship between self-employment and work-related stress (Hessels et al., 2017) shows that the findings based on HILDA can be generalized to other countries, in particular to countries with a similar income level per capita as Australia. Arguably, the same scope of generalization may hold for the results of the present study.

## Conclusion

Market dynamics make that the occupation of self-employed individuals is characterized by a relatively high level of uncertainty (Wennekers et al., 2007) and that the self-employed are often involved in a wide variety of tasks for which they are not well prepared (Baron, 2008). The self-employed often work long hours (Hyytinen and Ruuskanen, 2007) and perform a broad range of tasks to start and operate their business (Lazear, 2005). Therefore, several studies consider good health to be of utmost importance to adequately handle challenges, adversity, and stressors that come with being self-employed as well as to run a business successfully (Torrès, 2012; Rietveld et al., 2015; Hessels et al., 2018). Financial performance in self-employment depends heavily on the individual’s ability to work (Rietveld et al., 2015). In the present study, we assessed the relationship between an entrepreneur’s health status and his/her earnings. We provided robust evidence for a positive relationship between health and financial performance in self-employment in terms of earnings. All in all, this relationship can be considered to be a “double-edged sword” (Lewin-Epstein and Yuchtman-Yaar, 1991): good health boosts earnings in self-employment more than earnings in wage work, but ill health reduces earnings more substantially in self-employment compared to wage work.

The results of our study underscore the importance of health for the financial performance of the self-employed and their businesses. However, they also have implications beyond the individual level. The self-employed play an important role in the economy as job creators and innovators (Van Praag and Versloot, 2007; De Wit and De Kok, 2014). While companies often offer programs for improving health and vitality of their employees, the positive link of health with entrepreneurial earnings illustrates that it is of great importance to maintain and enhance the self-employed’s health as well. There may be a role for policy makers here, because occupational patterns in earnings dynamics may have detrimental effects on inequality (Castellano et al., 2019a, b). Although self-employment earnings are relatively high when being in good health, a deterioration of health is associated with a comparatively strong reduction in earnings and makes that individuals may have to abandon their business (a relationship for which we also provide evidence in the present study). Just as for other ingredients of human capital, such as cognitive ability and education, it seems “efficient” to have the healthiest individuals running their own business (Van Praag et al., 2013). However, the relatively transient nature of health compared to education and cognitive ability makes such a recommendation not entirely straightforward. Still, also in light of increasing numbers of self-employed individuals without employees in most developed countries (Van Stel and van der Zwan, 2019), we recommended policy makers to develop the social security system in such a way that it is sufficiently robust against a possibly increasing number of self-employed individuals in ill-health because the self-employed are often not covered by health insurance.

Although we provide novel evidence in this study about the stronger relationship between health and earning in self-employment than in wage work, we believe there are other important aspects of this relationship we left unaddressed in the present study. An important direction for future research may therefore be to assess how health develops over time in self-employment. The self-employment may be relatively healthy when starting a business, but health and associated feelings of vitality possibly differ depending on whether the entrepreneur has just started, whether he/she is experiencing difficulties with the firm, or whether the firm is growing. In some of these stages, good health may be more crucial than in others. Another direction for future research could be to evaluate the effect of health on other relatively objective performance indicators for self-employment such as business growth in terms of the number of employees, innovative capacity, as well as to assess the impact of entrepreneurs’ health on the performance and wellbeing of their employees. Healthy entrepreneurs have high energy levels and are mentally and physically vigorous. As such, their vitality may not only benefit themselves but may also have spillover effects on their employees. Finally, we used moderation analysis to study the relative impact of health on earnings in self-employment and wage work. More extensive mediated moderation analyses may be adopted in future studies to provide compelling empirical evidence about the precise mechanisms explaining the interaction effects found in the present study.

## Author’s Note

This manuscript uses unit record data from the Household, Income and Labor Dynamics in Australia (HILDA) Survey. The HILDA Project was initiated and is funded by the Australian Government Department of Social Services (DSS) and is managed by the Melbourne Institute of Applied Economic and Social Research (Melbourne Institute). The findings and views reported in this manuscript are those of the authors and should not be attributed to either DSS or the Melbourne Institute.

## Data Availability Statement

The dataset analyzed for this study can be obtained through https://melbourneinstitute.unimelb.edu.au/hilda/for-data-users.

## Ethics Statement

Ethical review and approval was not required for the study on human participants in accordance with the local legislation and institutional requirements. The patients/participants provided their written informed consent to participate in this study.

## Author Contributions

JH, CR, and PZ were involved in all parts of the study, except the data analysis. PZ performed the data analysis.

## Conflict of Interest

The authors declare that the research was conducted in the absence of any commercial or financial relationships that could be construed as a potential conflict of interest.

## Footnotes

1 The highest number of years of schooling completed is 8, 9, 10, 11, or 12 years. If an individual followed higher education, the values are recoded as follows: postgraduate degree (17), graduate diploma/certificate (16), bachelor’s degree (15), and advanced diploma/diploma/certificate (12).

2 Dummy variables for the following states have been included: Australian Capital Territory, New South Wales, Northern Territory, Queensland, South Australia, Tasmania, Victoria, and Western Australia.

3 Based on the Australian and New Zealand Standard Industrial Classification (ANZSIC).

4 Hausman tests also indicate that fixed-effects specifications are preferred over random-effects specifications.

5 For example, the coefficient of the interaction term *Health* × *Self-employment* is reduced by 34.7 and 21.9% for *General health* and *Physical health*, respectively. The coefficient of the interaction term *Health* × *Self-employment without employees* is reduced by 29.0 and 10.2%, respectively.

6 We consider someone experiencing a negative health shock if his/her value on the original 0–100 health scale is at least 75 in period *t* − 2, and below 75 in *t* − 1 and *t*; We consider someone not experiencing a negative health shock if he/she has a score of at least 75 at *t* − 2, *t* − 1, and *t*.
